# AirPredict: an eHealth platform for asthma management leveraging wearable sensors, digital diaries, and air quality monitoring to optimize patient outcomes

**DOI:** 10.3389/fdgth.2025.1573342

**Published:** 2025-06-06

**Authors:** Michele Atzeni, Luca Cossu, Sergio Gaiotti, Giacomo Cappon, Mariaenrica Tinè, Daniele Previtero, Ylenia Padrin, Simonetta Baraldo, Umberto Semenzato, Martina Vettoretti

**Affiliations:** ^1^ Department of Information Engineering (DEI), University of Padova, Padova, Italy; ^2^ Department of Cardiac, Thoracic, Vascular Sciences and Public Health, University of Padova, Padova, Italy

**Keywords:** asthma management, digital health, wearable sensors, air quality monitoring, feasibility study, air pollution, exposure, mobile application

## Abstract

**Introduction:**

Asthma management is complex, and while it is known that many environmental factors play a critical role in exacerbations, there is increasing interest on the role of air pollution. Yet, the precise relations by which these factors affect exacerbation risk are not fully understood. There is a need for innovative solutions to monitor and assess personal exposure to air pollutants in both outdoor and indoor environments to better understand their impact on respiratory outcomes, particularly asthma exacerbations. This paper introduces AirPredict, an innovative eHealth platform designed to enhance asthma management through the integration of wearable sensors, digital diaries, and ambient air quality monitoring.

**Methods:**

AirPredict comprises a mobile application for patients, a web interface for clinicians, and a robust cloud-based infrastructure. The platform utilizes devices such as the Fitbit Charge 6 for heart rate monitoring, the Atmotube PRO for air quality assessment, and the MIR SmartOne for spirometry, providing precise, real-time data on individual exposures and health outcomes. A feasibility study involving 16 participants, including asthma patients and specialized clinicians in Padova, Italy, was conducted to evaluate the usability of platform's components.

**Results:**

The results indicated high usability and user satisfaction, with average Single Ease Question (SEQ) scores ranging from of 6.8 to 5.5 out of 7 for patients and from 6.8 to 6.6 for clinicians, reflecting ease of use and functionality.

**Discussion:**

The findings support the platform's broader adoption and further development, highlighting its role in advancing eHealth solutions for chronic disease management.

## Introduction

Asthma is a prevalent and heterogeneous chronic respiratory disease that affects 1%–29% of the global population, varying among different countries (1). It manifests through symptoms such as wheezing, shortness of breath, chest tightness, and coughing, alongside variable expiratory airflow limitation. Both symptoms and airflow limitation characteristically vary in severity and duration. These variations are often influenced by different triggers, such as exercise, allergens, viral infections, or environmental factors like weather changes and air pollution. Effective management is crucial as asthma exacerbations can carry a significant burden to patients and the community, leading to hospitalizations, reduced quality of life, loss of work/school days, and fatalities (1).

Air pollution, particularly small particles and gases, poses significant health risks. Urban areas with dense populations and vehicular emissions have elevated levels of pollutants like particulate matter (PM1.0, PM2.5, and PM10), ozone (O3), and nitrogen dioxide (NO2). Particulate matter, especially PM2.5, can penetrate deeply into the lungs and enter the bloodstream, while ground-level ozone and nitrogen dioxide, both components of smog, contribute to asthma symptom persistence and exacerbations.

Monitoring environmental exposures and their health impacts requires timely and appropriate information for public health planning and surveillance. Most studies have examined the impact of environmental exposures on asthma at the population level (2), with only a few studies focusing on individual-level assessments, which can reduce confounding factors (3, 4). With the rapid proliferation of mobile devices and advanced sensors, there is growing interest in using wearable sensors to quantitatively define individual exposure to environmental pollution (5–9). These devices can provide real-time feedback on environmental exposures, enabling users to make informed decisions to minimize their risk. Research has demonstrated the efficacy of wearable sensors in assessing air quality, highlighting significant variations across different locations.

Despite the potential of wearable sensors, there remains a gap in integrating these devices with platforms that collect and analyze patient-specific respiratory outcomes. Existing eHealth platforms often focus either on patient symptom tracking or environmental exposure monitoring, rarely both (10). Nevertheless, the integration of personal exposure to air pollution data with individual respiratory outcomes is crucial for studying the impact of personal exposure to air pollution on asthma outcomes.

To fill this gap, in this work we present AirPredict, a sensor rich General Data Protection Regulation (GDPR) compliant platform that will support research on the impact of personal exposure to air pollution on respiratory outcomes in asthmatic individuals. A smartphone is used as a hub for collecting physiological, environmental, and human input questionnaire data. Physiological information includes respiratory function measurements, collected by a portable spirometer, and heart rate data, collected by a smartwatch; environmental data includes air pollution measurements, collected by a wearable air quality sensor; and human input questionnaire data includes information about symptoms, exacerbations, and asthma status control, logged by the patient through the app. This information is then pushed to a cloud database and remotely accessible from physicians and researchers through a Web interface.

Unlike conventional asthma management tools, which primarily focus on health metrics such as peak flow measurements or medication adherence, AirPredict also continuously monitors environmental factors, such as air pollution levels, alongside health data. This integration will provide more precise, context-aware information into how environmental exposures may affect asthma control in near real-time.

The AirPredict platform is potentially useful for three categories of users: patients, physicians and researchers. For patients, it provides the ability to accurately assess their individual PM exposure, as well as log and track essential clinical and functional data daily. The platform will allow physicians to remotely monitor both the environmental exposure and the respiratory symptoms of their patients, potentially allowing them to identify environmental triggers of asthma symptoms and suggest preventive measures; furthermore, the platform provides a method for early detection and precise quantification of asthma exacerbations and oral corticosteroid use. Finally, the platform is valuable for researchers because it allows to collect rich datasets, integrating both exposure to air pollution and respiratory outcomes data, that can be exploited to investigate the impact of air pollution on asthma exacerbations. In particular, such datasets will allow to develop predictive models of asthma exacerbations that leverage information of the personal exposure to air pollution.

The platform has been evaluated through a 30-day experiment in which users were asked to complete some tasks and then rate the usability via the Single Ease Question (SEQ) and System Usability Scale (SUS) questionnaires for data-driven User eXperience (UX) evaluation.

## Methods

### Platform architecture

**Figure 1:**
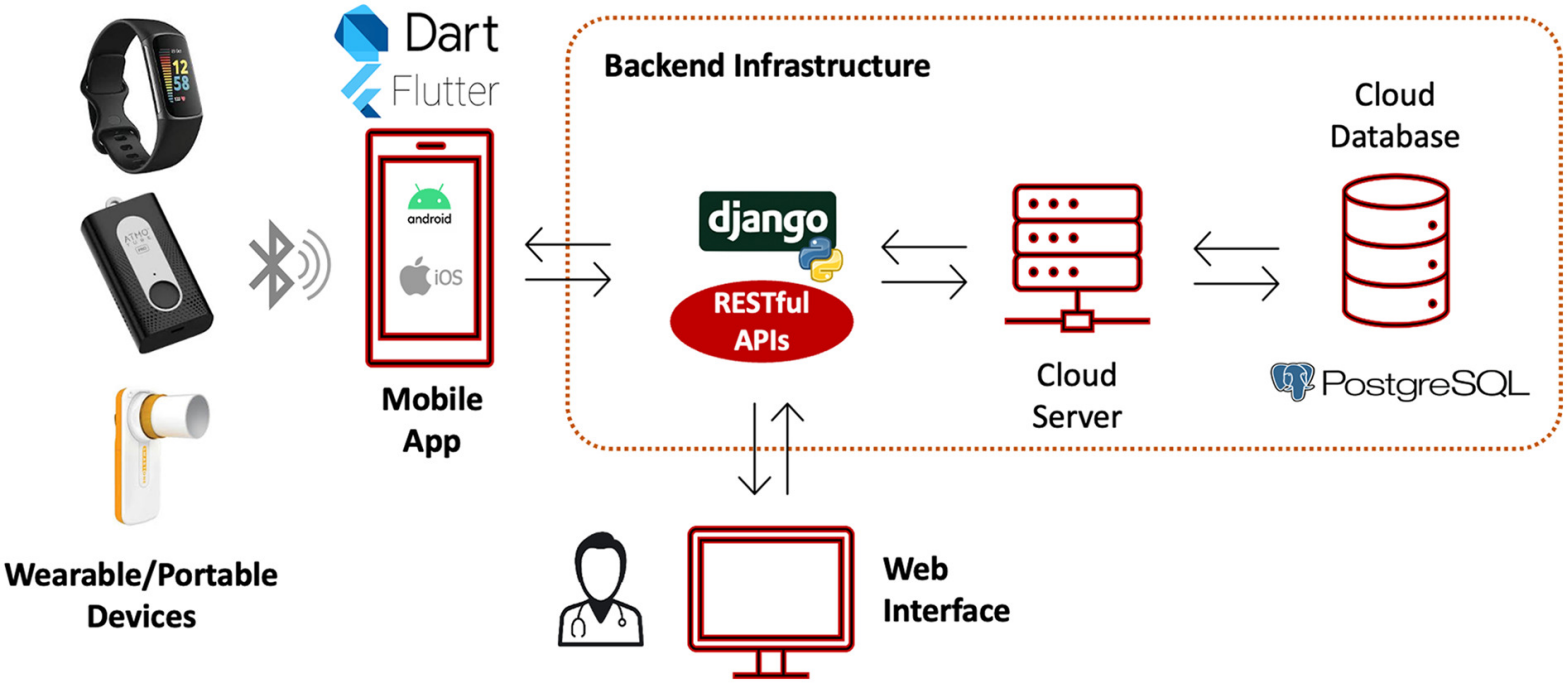
Platform overview. The mobile app collects data from patients’ wearable/portable sensors, as well as data manually logged by the patient, and stores them in the cloud database through ad-hoc RESTful APIs. The cloud database is also queried by a clinical web interface that allows physician to monitor in real-time their patient data and insert data during the in-hospital visits. Icons/images reproduced with permission from; “Bluetooth icon” by Maan Icons, “Dart” logo, “Flutter” logo, “PostgreSQL” logo, Fitbit charge 6, Atmotube Pro, MIR Smart One. “Django” logo created using “Python-logo-notext” by http://www.python.org/, licensed under GNU General Public License and from https://www.djangoproject.com/.

As illustrated in Figure 1, the platform is structured around four core components:

1. Wearable or portable devices. These devices are compact technologies used for real-time monitoring of health metrics (i.e., heart rate and respiratory function parameters), or environmental data (i.e., air quality indices). In particular, a wearable air quality sensor allows to measure air quality at the patient location (both outdoor and indoor) in dynamic settings (e.g., commuting).
2. Mobile Application. This component is designed for automated data collection from integrated sensors. Additionally, it provides an intuitive interface for patients to log daily spirometry data, exacerbation events or assess the status of asthma control.
3. Web Interface. Aimed at healthcare professionals, this interface allows to register and enroll asthmatic patients in the monitoring programme, and to remotely review and analyze individual patient data in real-time.
4. Backend Infrastructure. This component, composed of the RESTful API, cloud server and cloud database, ensures the safe storage of collected data and manages the secure transmission of data between the different platform's components.

To ensure the confidentiality and integrity of the data, the platform incorporates Role-Based Data Access Control (RDAC). RDAC restricts access to sensitive data based on the user's role within the system, ensuring that only authorized personnel, such as patients, healthcare providers or researchers, can access specific information based on predefined permissions. The communication between the platform components is protected with strong authentication protocols, including Jason Web Token (JWT) authentication for secure login mechanisms, and uses Hypertext Transfer Protocol Secure (HTTPS) for encrypted communication.

### Wearable or portable devices

**Figure 2:**
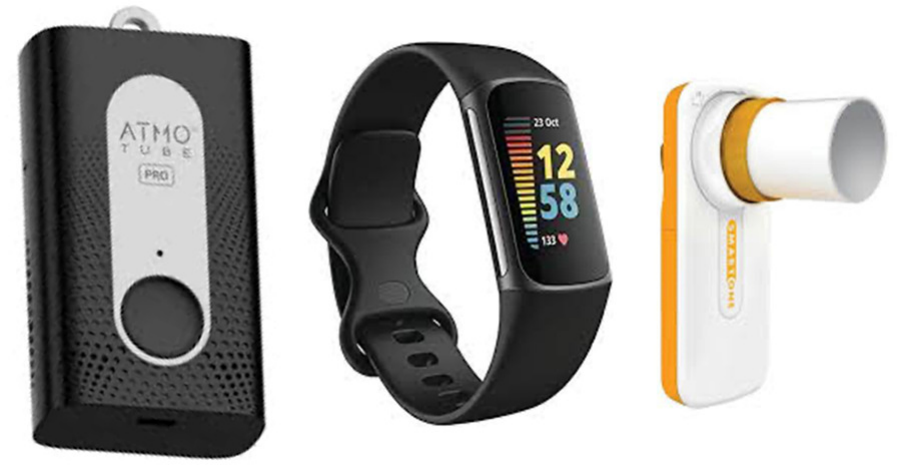
Sensors integrated in the platform. From the left: Atmotube PRO, wearable air quality sensor; Fitbit Charge 6, wristwatch; MIR SmartOne, portable spirometer. Atmotube Pro, Fitbit charge 6 and MIR Smart One reproduced with permission.

To assess the personal exposure to air pollution and respiratory function, the platform gathers data from 3 wearable/portable sensors: the Atmotube PRO (Atmotech Inc., CA, USA), a wearable air quality sensor, the Fitbit Charge 6 wristwatch (Fitbit Inc., CA, USA), and the MIR SmartOne (MIR S.p.A., Rome, Italy), a portable spirometer (Figure 2). In the selection of wearables, factors such as cost, performance, and ease of data access were evaluated to guarantee the optimal choice for monitoring both health and air pollution.

The Atmotube PRO, hereinafter referred to as Atmotube, is a Bluetooth connected wearable device that detects volatile organic compounds (VOCs), particulate matter (PM) of various size (1.0, 2.5 and 10 µg), temperature and humidity levels (11, 12). The device employs an optical PM sensor based on the laser light scattering principle (13). PM measurements are collected at almost continuous time, with sampling period that can be set to always on, 5, 10, or 15 min. Before taking measurements, the device actively draws in air through an internal fan (13). A recent field evaluation conducted by the South Coast Air Quality Management District (SCAQMD) affirmed the Atmotube's high accuracy in detecting ambient PM1 and PM2.5 concentrations, showing a strong correlation with Federal Equivalent Method (FEM) instruments (R^2^ values ranging from 0.79 to 0.94) (11).

The Fitbit Charge 6 is a wrist-worn Bluetooth connected device designed to track health and physical activity metrics. Heart rate data is obtained through photoplethysmography (PPG), which employs a light source and a photodetector on the skin's surface to gauge blood flow variations by detecting the amount of absorbed light. Heart rate data are collected at almost continuous time, with a sampling period of 5–15 s. Heart rate data are important for accurate estimation of the personal exposure to air pollution as, through suitable mathematical models, they can provide indirect estimates of the patient ventilation rate (14), which allows to estimate the amount of inhaled pollutant dose (15).

MIR Smart One is a pocket-sized, Bluetooth connected, personal spirometer that measures the Peak Expiratory Flow (PEF), i.e., a measure indicating the maximum volume of air expelled with maximal effort, and Forced Expiratory Volume in 1 s (FEV1), i.e., the volume of air exhaled in the first second during forced exhalation after maximal inspiration.

Apart from MIR Smart One, whose data is logged manually in the App, the Fitbit and Atmotube PRO devices are connected through dedicated APIs that allowed their integration with the AirPredict app. The Fitbit API provides access to various health-related data, with a primary focus on heart rate, which is essential for evaluating exposure to air pollution. The AirPredict app regularly fetches this data from the Fitbit API using secure OAuth 2.0 authentication each time the patient logs into the application, so that the data is updated in near real-time.

The Atmotube API, on the other hand, enables the fetch of ambient air quality measurements at regular intervals. This integration is granted by Atmotech, who provides a unique API Key upon registering the sensor's serial number in their systems.

### Mobile application for patients

The AirPredict mobile application is developed in Flutter (version 3.3.8) (16) and is cross-platform, hence targeting devices embedded with iOS or Android. The app provides patients with a quantitative assessment of their daily PM exposure, and allows the logging of health metrics through structured questionnaires grouped within an “Asthma Diary” module. These questionnaires include:

1. Asthma Control Test (ACT). The ACT is a patient self-administered tool for identifying those with poorly controlled asthma. It is a 5-item, with 4-week recall questionnaire which assesses the frequency of shortness of breath and general asthma symptoms, use of rescue medications, the effect of asthma on daily functioning, and overall self-assessment of asthma control (17).
2. Spirometry and time spent outdoors (Daily Track). This questionnaire collects daily information about:
  a. PEF and FEV1 measurements collected by the MIR Smart One;
  b. asthma medication taken in the previous 6 hours;
  c. time spent outdoors during the day (6:00–21:00) and night (21:00–6:00).
3. Exacerbation Report (Asthma Attack). This module enables users to document episodes of asthma exacerbation, detailing the severity, associated triggers, and pharmacological interventions employed.

**Figure 3:**
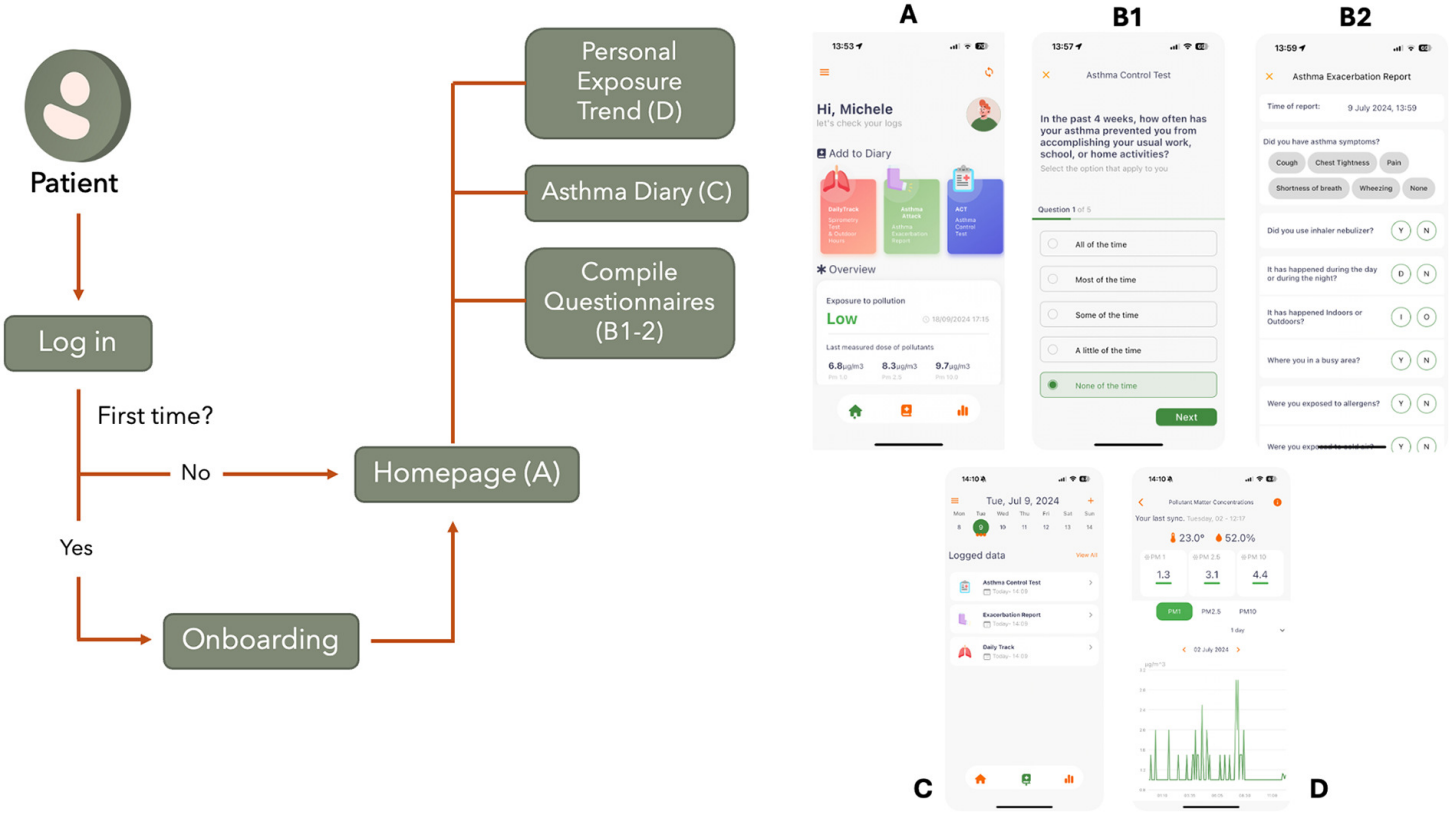
Main schema and screenshots of the AirPredict mobile application. Patients log in the app. If it is the first time, they are redirected to an onboarding page to log useful profile information. The homepage **(A)** allows to collect asthma related information **(B1–2)** and check personal exposure to pollutants. The asthma diary **(C)** let the user see the compiled asthma questionnaires at a glance. The personal exposure trend section **(D)** let the user see the daily, weekly and monthly trends of PM timeseries. Screenshots from AirPredict app.

Figure 3 shows the application flow schema and screenshots of the main features. Only verified users can access the mobile application, which ensure confidentiality and data integrity. If it is the first time that a user logs in the application, an onboarding step is mandatory. The onboarding allows collecting informative data for exposure estimation (e.g., biological sex, age, resting heart rate) and make the necessary connections and authorization to gather wearable sensors’ data. Furthermore, the onboarding explains the functionality of each provided sensor and their scope. Otherwise, the user is redirected to the home page, which displays the last PM measurements recorded by the air quality sensor and provides quick access to the asthma diary questionnaires. From the homepage, the Asthma Diary, and Exposure Page can also be explored. The first one displays the past logs and allows to log new data through the questionnaires, while the latter displays PM concentrations, and activity trends (heart rate and steps timeseries).

To enhance user adherence and consistent data input, the application is equipped with customizable reminders. Design considerations have been made to ensure data integrity, accessibility, privacy, and GDPR compliance, to protect and to accommodate a diverse user base with varied technical proficiencies.

To address potential gaps in environmental data, a GPS location is recorded every 20 minutes to potentially correlate with fixed monitoring station data and evaluate personal exposure. To protect privacy, the platform does not store exact GPS coordinates, but only the Zone Improvement Plan (ZIP) code of the patient location.

Access to the system is granted only to patients who have provided informed consent in advance. Data in transit is protected by JWT authentication, whilst data at rest (in-App database) is encrypted with the 256-bit Advanced Encryption Standard (AES) and protected by password. All the communications are encrypted via the HTTPS protocol. Finally, to comply with data protection regulations, the platform follows the principles of sensitive data minimization and pseudo-anonymization (i.e., users are identified only by a random alphanumeric code). Moreover, patients retain full control over their personal data, with the right to access, rectify, and request the deletion of their information at any time of the study.

### Web interface

**Figure 4:**
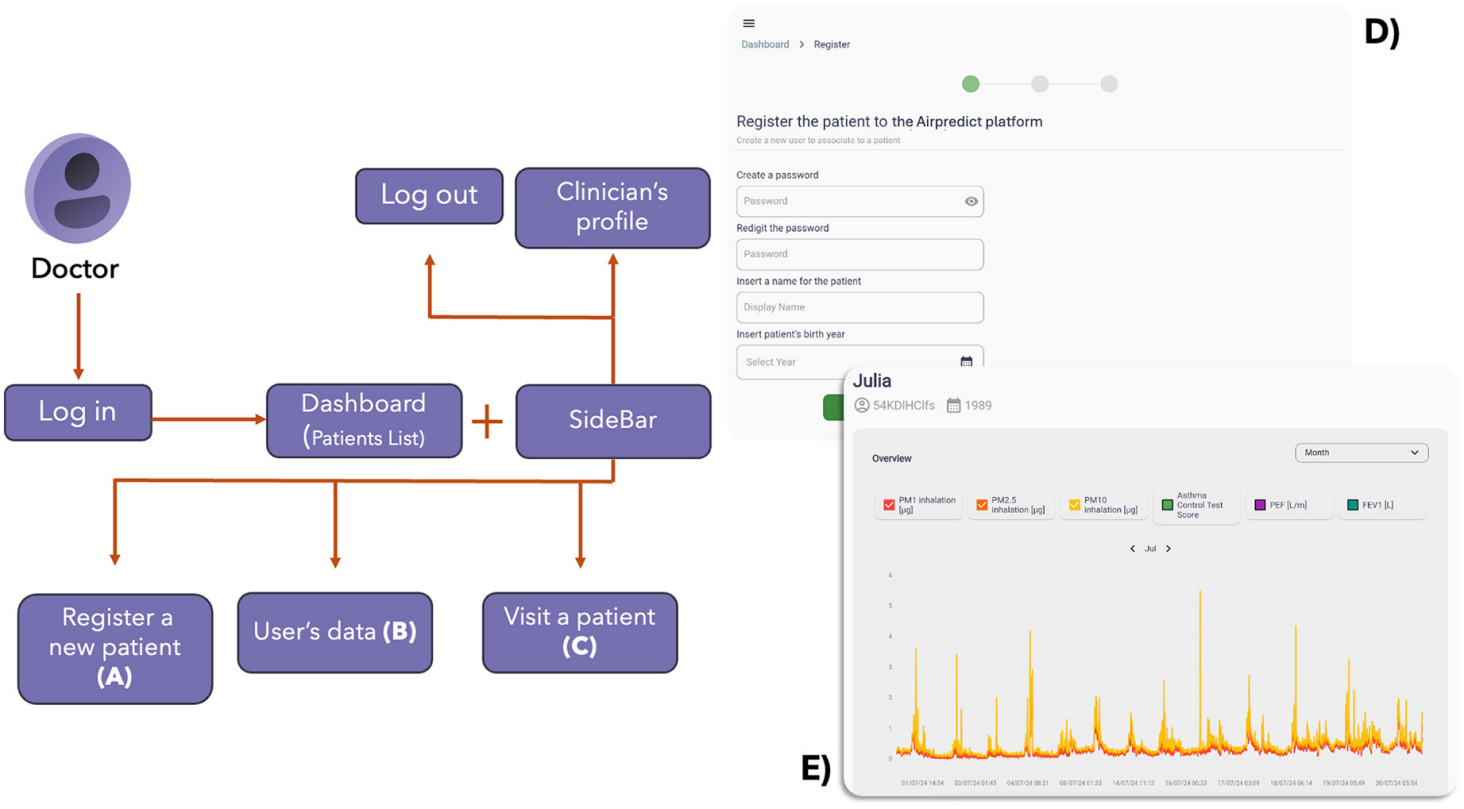
Main schema and screenshots of the AirPredict web interface. A clinician logs in the app. The homepage allows to see all the enrolled patients in the monitoring programme. From here, a new patient can be enrolled filling the necessary fields **(A)**, or a visit can be done **(C)** Moreover, for each patient, their trends and asthma status can be checked **(B)** visualizing plots and questionnaires answers. **(D)** and **(E)** are screenshots of the interface, respectively about the **(A)** and **(B)** functionalities. Screenshots from AirPredict website.

To assist clinicians and providers in organizing user registrations and remotely monitor the data of their patients, a web interface has been developed. The web interface (developed using Flutter 3.3.8) (16) will allow access to patient data collected throughout the study, which is then restricted only to authorized healthcare professionals (with JWT). Moreover, as for the mobile app, data transmission is encrypted (HTTPS protocol). Post-authentication, healthcare professionals are presented with an overview of only the data related to their patient group and can access to several functionalities (Figure 4).

This web interface provides a snapshot of the enrolled patients, facilitating quick navigation and management. One of the primary functionalities is the ability to register new patients in the monitoring programme. This module allows for the input of essential patient demographics, medical history, and other pertinent details, via enrollment questionnaires. The interface also allows to insert new data during follow-up visits by follow-up questionnaires. This feature ensures that each patient's data is recorded and timestamped, facilitating the creation of longitudinal patient data. Furthermore, for each patient, the platform provides detailed data visualizations. This includes:

1. Exposure Trends. Graphical representations of the patient's environmental exposure and health status data (PM 1, 2.5 and 10, ACT scores, PEF, and FEV1), offering insights into potential triggers and patterns (Figure 4E).
2. Self-Reported Questionnaires. Logs of patient-reported outcomes (Daily Track, Asthma Attack, and ACT), visualized over time to track symptom progression or improvement.
3. Enrollment/Follow-Up Questionnaires Outcomes. A repository of data collected by the enrollment and subsequent follow-up questionnaires, allowing for a longitudinal view of key patient's health data.

### Backend and cloud database: impact 2.0

The backend infrastructure includes a cloud server, hosting the database, communicating with the mobile app and the web interface through a RESTful web API. The backend infrastructure is an extension of an established platform, previously developed by the Bioengineering group at the Department of Information Engineering (DEI) of the University of Padova, namely IMPACT. This platform, originally conceptualized for the surveillance of diabetic patients (18), has an inherent adaptability, making it customizable for the current research objectives. While the primary monitoring targets differ, the main architecture and its inherent robustness remain consistent. It has been developed using Django, a Python framework for backend web applications that encourages rapid development and clean, pragmatic design.

All collected data is stored securely on a local server at DEI, University of Padova (Padova, Italy) with security and access control mechanisms in place.

### Feasibility study

The AirPredict platform was tested in a feasibility study involving 16 volunteers from the University of Padova (Padova, Italy): 8 asthma patients and 8 specialized asthma-monitoring clinicians. The study lasted 4 weeks and included three key phases: enrollment, daily monitoring, and final assessment.

During the initial visit, each clinician enrolled a patient and registered them in the study. Prior to receiving any training or further explanation about the platform, both clinicians and patients independently completed some usability tasks (Table 1) on the web interface and the mobile application, respectively. After completing the test, patients were provided with three devices: a SmartOne spirometer, an Atmotube PRO air quality monitor, and a Fitbit Charge 6 smartwatch. They were instructed to install and configure the AirPredict, Atmotube, Fitbit, and SmartOne applications on their smartphones with the help of an engineer. Post-enrollment, patients were required to use the provided devices daily, ensuring they were charged and synchronized with the respective applications at least once a day. Every evening, patients conducted a spirometry test using the SmartOne spirometer, and recorded the FEV1 and PEF values, along with filling information about medication use, and time spent outdoors, in the AirPredict app via the Daily Monitoring questionnaire. Additionally, patients filled out a bi-weekly ACT questionnaire through the app. In case of an asthma exacerbation, they submitted an Exacerbation report through the app. Finally, patients compiled a form giving feedback about the whole infrastructure, reporting any issues with the applications, devices, or study procedures to the engineering team, allowing for the identification of bugs, feature improvements, and any difficulties encountered during the study.

**Table 1:** Tasks description, scenario and solution for the web dashboard and mobile application. Tasks were ten in total, five for each product.

A. AirPredict clinical dashboard (web interface)			
Task ID	Task description	Scenario	Navigation path
1	Register a patient	Imagine you want to register a new patient; how would you navigate within the homepage?	Navigate to the patient registration section from the homepage.
2	Perform the first visit of the patient after registration	Imagine you want to perform the first visit of the newly registered patient; how would you navigate?	Navigate from the homepage to the patient's first visit section.
3	Complete questionnaires for a follow-up visit	Imagine you want to complete the questionnaires for a follow-up visit of test patient, how would you navigate?	Navigate to the follow-up questionnaires section from the homepage.
4	View personal exposure and activity of a patient	Imagine you want to check the PM2.5 concentration of test patient in May, how would you navigate?	Navigate to the personal exposure and activity section from the homepage.
5	View the progress of asthma control tests of a patient	Imagine you want to check the progress and details of asthma control tests of test patient in May, how would you navigate?	Navigate to the asthma control test details section from the “Patient Overview” page.
6	Register a patient	Imagine you want to register a new patient; how would you navigate within the homepage?	Navigate to the patient registration section from the homepage.
B. AirPredict mobile application			
Task ID	Task description	Scenario	Navigation path
1	Complete the daily spirometry monitoring questionnaire and asthma control Questionnaires	Imagine you want to enter the results after using the SmartOne spirometer, fill out the spirometry questionnaire, and note outdoor time (or record an asthma attack, or conduct an asthma control test). How would you navigate within the application?	Navigate from the homepage to the daily track (or to the asthma attack questionnaire or to the ACT questionnaire).
2	View daily logs	Imagine you want to check the data you just entered through the questionnaire; how would you navigate from the homepage?	Navigate from the homepage to the Asthma Diary section.
3	View charts related to personal exposure to pollutants	Imagine you want to check the chart for personal PM1.0 exposure from the last day, how would you navigate from the homepage?	Navigate from the homepage to the Personal Exposure Trends section.
4	Check the dose of pollutants	Imagine you want to check the latest daily measurement of PM2.5 pollutants, how would you navigate within the application?	Navigate from the homepage to the Pollutant Matter Measurements section.
5	Synchronize data	Imagine you want to synchronize your data with the physician, how would you navigate within the application?	Tap the synchronization icon on the top left in the homepage.

**Figure 5:**
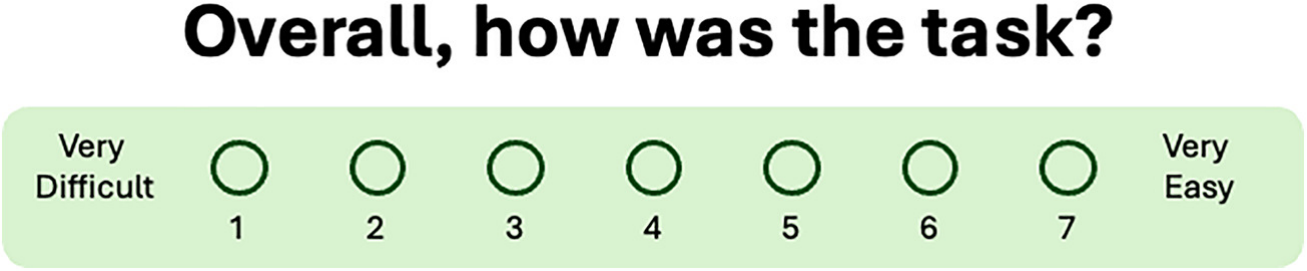
Single ease question to ask after each administered task. The 7-point rating scale goes from very difficult—1—to very easy—7.

Usability testing for the web application required physicians to perform tasks such as registering a patient, conducting the first patient visit, filling out follow-up visit questionnaires, and viewing patient exposure and activity data (Table 1A). For the mobile application, patients were tasked with completing daily spirometry monitoring, asthma control tests, viewing daily logs, pollution exposure graphs, and synchronizing data with their physicians (Table 1B). The Single Ease Question (SEQ) (Figure 5) questionnaire was administered after each task during the initial visit to evaluate the ease of task completion. The SEQ is a 7-point rating scale to assess how difficult users find a task (19). Additionally, the total time taken to complete all five tasks, along with participants’ age, digital literacy (as the ability to effectively use digital tools and technologies, measured on a scale from 0 to 5), and education level were recorded.

At the end of the study, all participants completed the System Usability Scale (SUS) questionnaire to provide a comprehensive evaluation of the platform's usability. The SUS is a 10-item attitude Likert scale questionnaire that allows to collect a global view of subjective assessments of usability (21). Results from the usability tests were analyzed and visualized in Python 3.9 (22).

## Results

During the first visit and testing phase, baseline data were collected to record the main characteristics of the study participants, providing information about demographics and digital literacy. The eight patients had a median age of 29.5 years, with 50% females and a median digital literacy of 4.5 out of 5. On the other hand, clinicians had a median age of 29.5 years, with 89% females and a median digital literacy of 3 out of 5. All the participants are graduates.

**Figure 6:**
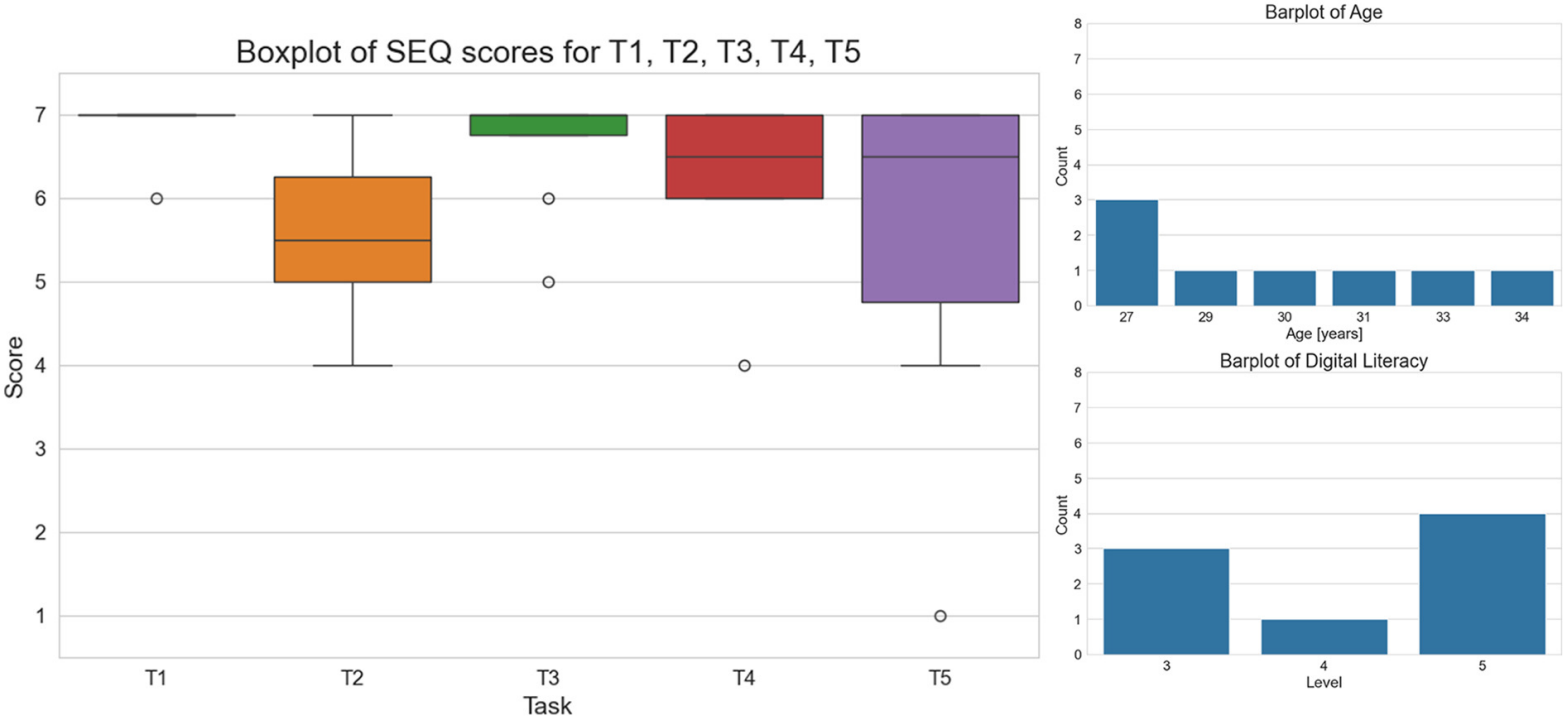
On the left, boxplot of the SEQ scores for each task. On the right, digital proficiency and age distribution of the patients involved in the usability test.

**Figure 7:**
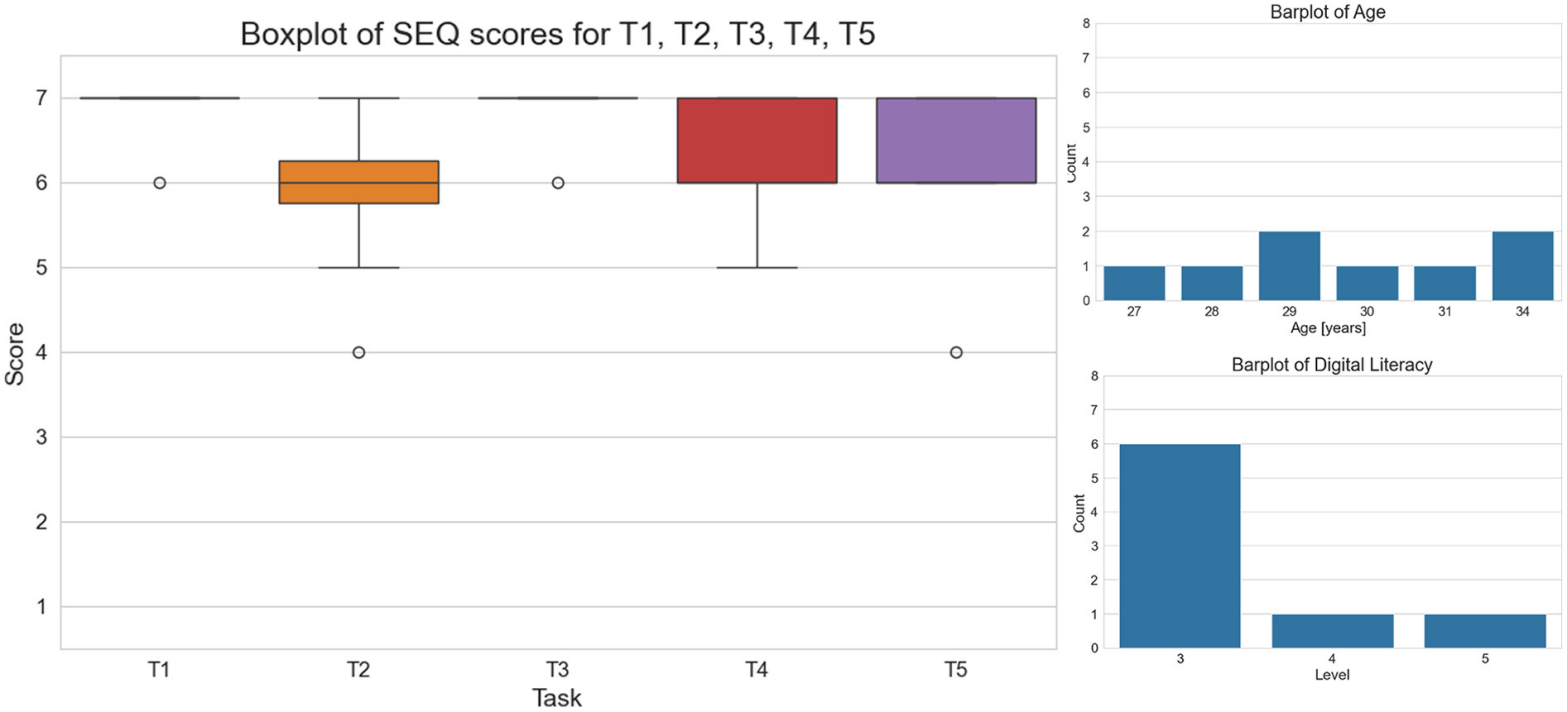
On the left, boxplot of the SEQ scores for each task. On the right, digital proficiency and age distribution of the clinicians involved in the usability test.

The results, as shown in Tables 2, 3 and Figures 6,7, present both the mean and the standard deviations of the SEQ scores for each task, as well as a boxplot representation of their distribution.

**Table 2:** Mean and 95% confidence intervals (C.I.) of the SEQ scores, and of the time in minutes for all patient testers involved in the usability test.

Task ID/total time	Mean [C.I.]
T1	6.87 [6.58–7.00]
T2	5.62 [4.74–6.51]
T3	6.62 [6.00–7.00]
T4	6.25 [5.38–7.00]
T5	5.5 [3.71–7.00]
Total time [min]	4 [2.84–5.31]

**Table 3:** Mean and 95% confidence intervals (C.I.) of the SEQ scores, and of the time in minutes for all clinician testers involved in the usability test.

Task ID/total time	Mean [C.I.]
T1	6.87 [6.58–7.00]
T2	5.87 [5.05–6.70]
T3	6.87 [6.58–7.00]
T4	6.25 [5.66–6.84]
T5	6.12 [5.29–6.95]
Total time [min]	17.5 [15.19–20.30]

Overall, the mobile app has been perceived as very easy to use (Table 2 and Figure 6). Task 1 (T1) and Task 3 (T3) were generally perceived as the easiest tasks (average SEQ scores of 6.87 and 6.62, respectively), with high consistency among users [95% confidence intervals of SEQ scores: [6.58–7.00] and [6.00–7.00]], suggesting they are well-designed and intuitive. Although tasks T2, T4 and T5 were generally perceived as easy, a greater variability in perceived ease was observed. The variability in total time taken, 4 (1.5) minutes, also highlights the need to consider different user experiences and proficiencies.

The predominance of high literacy levels among subjects likely contributed to the generally high SEQ scores, as these individuals may find it easier to navigate and understand the tasks. However, the variability in task ease suggests that even among a literate group, task design can significantly impact user experience. The age distribution, in the range (27, 34) years, suggests that the subjects are relatively young, possibly correlating with a higher comfort level with technology. However, the presence of variability in SEQ scores, especially for Task 5, indicates that age alone does not account for all differences in user experience, emphasizing the importance of task design in usability studies (Figure 6).

The SEQ scores for tasks performed by clinicians with the web interface reveal a generally high perception of ease for most tasks, similar to the mobile application feedback (Table 3 and Figure 7). Tasks 1 and 3 were particularly well-received, with high mean scores and low variability, indicating effective and intuitive designs. Task 2 and Task 4 showed moderate ease with greater variability, suggesting areas where usability could be enhanced to achieve more consistent user experience. Task 5, while still relatively easy, had the lowest mean score and highest variability, highlighting it as a potential focus for improvement.

The total time for completing the tasks suggests that completing all tasks required a significant amount of time, with notable differences between users. This variability can be attributed to the complexity of Task 2, which involves the enrollment visit for a patient and requires completing several questions.

Declared digital literacy level among clinicians was lower compared to the mobile app users (distribution skewed towards a score of 3). Regardless the overall lower literacy, the SEQ scores were quite high, suggesting that the web interface is overall clear and very easy to use. The age distribution suggests that the subjects are older than the mobile app users, but still belonging to generations very used to digital tools. This also potentially confirms the correlation with the comfort level with technology declared in both groups (Figure 7).

According to the SUS scores collected at the end of the study, clinicians rated the web interface very positively, with an average score of 81 (SD = 20), which highlights the effectiveness of the web interface that appears simple, intuitive and well aligned with their workflow and requirements. Patients, who evaluated the mobile application alongside the integration with the wearable sensors (Fitbit, Atmotube, and MIR SmartOne), provided an average score of 63 (SD = 18), indicating a marginally acceptable level of usability for the mobile system. Patients’ lower scores reflect the complexity of using multiple monitoring devices and synchronizing their data. Nevertheless, many patients provided positive feedback on the integration of various functionalities when everything worked as intended. However, synchronizing data from the sensors was reported as the most problematic task, especially for the Atmotube sensor, and consequently patients had difficulties in visualizing air quality data in the AirPredict application. It is important to consider these challenges for future improvements of the platform, in particular to enhance the data transmission. Despite these difficulties, users recognized that with regular use, the system became more familiar, suggesting that additional guidance or training could further enhance the user experience and ease of interaction.

## Discussion

The control of asthma, a prevalent chronic respiratory disease, is significantly influenced by environmental factors such as air pollution. Traditional asthma management approaches often overlook the potential negative impact of personal exposure to air pollution on respiratory outcomes at an individual level. The AirPredict platform represents a significant step forward in this direction, providing a system to monitor both asthma patients and their living environment, thus potentially providing relevant insights about the impact of personal exposure to air pollution on asthma outcomes. This work presented the development, implementation, and evaluation of AirPredict, highlighting its novel approach to asthma management through real-time, individualized environmental exposure and health outcome tracking.

AirPredict has been designed with cost-effectiveness, accessibility, and scalability in mind. By utilizing commercially available wearables and open-source development frameworks, the system remains financially viable for widespread adoption. Its cross-platform mobile compatibility, along with the web interface for clinicians, ensures accessibility for patients, accommodating individuals with varying levels of digital literacy, and healthcare providers, extending the platform's utility to clinical settings without requiring frequently in-person visits. The platform leverages wearable devices such as the Fitbit Charge 6 for heart rate monitoring and the Atmotube PRO for air quality measurements. Additionally, spirometry measurements are collected using the MIR SmartOne device. It is structured around four core components: usage of wearable/portable devices, a mobile application for patients, a web interface for healthcare professionals, and a backend infrastructure for secure data storage and transmission. The mobile application provides an intuitive interface for patients to log daily health metrics and view their exposure to pollutants. The web interface offers healthcare professionals a comprehensive dashboard to manage patient data, track exposure trends, and analyze self-reported health outcomes.

To assess the reliability and the efficacy of the platform, a feasibility study was conducted and involved 16 participants, including asthma patients and specialized clinicians. Over a four-week period, the study evaluated the platform via the SEQ and SUS questionnaires, along with additional feedback from the users. Results from the SEQ and SUS questionnaires indicated high user satisfaction, with average scores reflecting ease of use and functionality. The usability test demonstrated that both patients and clinicians found AirPredict highly usable and beneficial. Patients reported feeling more in control of their condition due to the detailed insights into their environmental exposures and health metrics. Clinicians appreciated the ability to correlate environmental data with patient symptoms, enabling more informed and proactive management strategies.

We acknowledge that the small sample size could limit the findings of this feasibility study, as it may not fully capture the diversity of user experiences across a broader population. While these findings provide initial evidence of the platform's reliability and usability, larger-scale studies are needed to validate these results further and assess the long-term engagement, as well as the effectiveness of AirPredict in routine asthma management. To address this, the enhanced platform will be used in an observational study involving 100 asthmatic patients as part of the BREATHE project, whose goal is to study the impact of personal exposure to air pollution on asthma exacerbations.

Although the system was generally accepted, mobile users proposed several enhancements to improve the UX and functionality of the platform. The evaluation revealed usability and technical challenges reported by users, primarily related to data synchronization app stability, and multi-device integration. A common issue was the frequent loss of synchronization, requiring app restarts to establish a proper connection. Therefore, these challenges were important areas of focus for subsequent iterations; in fact, an optimized synchronization process has been implemented, specifically to improve data transmission. The updated version includes features such as a sped-up data synchronization, and improvements were made to enhance the synchronization process with the Atmotube sensor, making it clearer and more responsive to reduce users’ uncertainty about its functionality. Additionally, a downloadable guide was made available to help users tackle synchronization issues effectively [download available at (23)]. Moreover, maintaining sustained engagement remains a challenge in digital health systems. Differences in technological literacy among users may influence adherence, and the repetitive tasks along with the continuous usage of the devices will lead to user fatigue. To address these challenges, future versions of AirPredict could incorporate enhanced user engagement strategies, such as gamification elements, to promote continued use. Further research will assess long-term adherence trends and refine the platform to optimize user retention.

In conclusion, the AirPredict platform, thanks to the integration of wearable sensors, a mobile app and a web interface for real-time data monitoring, will provide a significant advantage over traditional asthma monitoring systems. The ability to track personal exposure to pollutants and its impact on health outcomes will allow for a deeper understanding of triggers of asthma exacerbations. This comprehensive platform demonstrates the potential of eHealth solutions in chronic disease management, leading the way for improved patient monitoring and advancing public health.

## Data Availability

The raw data supporting the conclusions of this article will be made available by the authors, without undue reservation.
